# Level of Satisfaction of Older Persons with Their General Practitioner and Practice: Role of Complexity of Health Problems

**DOI:** 10.1371/journal.pone.0094326

**Published:** 2014-04-07

**Authors:** Antonius J. Poot, Wendy P. J. den Elzen, Jeanet W. Blom, Jacobijn Gussekloo

**Affiliations:** Department of Public Health and Primary Care, Leiden University Medical Center, Leiden, the Netherlands; Supportive care, Early Diagnosis and Advanced disease (SEDA) research group, United Kingdom

## Abstract

**Background:**

Satisfaction is widely used to evaluate and direct delivery of medical care; a complicated relationship exists between patient satisfaction, morbidity and age. This study investigates the relationships between complexity of health problems and level of patient satisfaction of older persons with their general practitioner (GP) and practice.

**Methods and Findings:**

This study is embedded in the ISCOPE (Integrated Systematic Care for Older Persons) study. Enlisted patients aged ≥75 years from 59 practices received a written questionnaire to screen for complex health problems (somatic, functional, psychological and social). For 2664 randomly chosen respondents (median age 82 years; 68% female) information was collected on level of satisfaction (satisfied, neutral, dissatisfied) with their GP and general practice, and demographic and clinical characteristics including complexity of health problems. Of all participants, 4% was dissatisfied with their GP care, 59% neutral and 37% satisfied. Between these three categories no differences were observed in age, gender, country of birth or education level. The percentage of participants dissatisfied with their GP care increased from 0.4% in those with 0 problem domains to 8% in those with 4 domains, i.e. having complex health problems (p<0.001). Per additional health domain with problems, the risk of being dissatisfied increased 1.7 times (95% CI 1.4–2.14; p<0.001). This was independent of age, gender, and demographic and clinical parameters (adjusted OR 1.4, 95% CI 1.1–1.8; p = 0.021).

**Conclusion:**

In older persons, dissatisfaction with general practice is strongly correlated with rising complexity of health problems, independent of age, demographic and clinical parameters. It remains unclear whether complexity of health problems is a patient characteristic influencing the perception of care, or whether the care is unable to handle the demands of these patients. Prospective studies are needed to investigate the causal associations between care organization, patient characteristics, indicators of quality, and patient perceptions.

## Introduction

Patient satisfaction, also referred to as global rating of health care, has an important but ambiguous role in patient-centered care [1]. Satisfaction is related to quality of service but not directly with quality of care [2]. Satisfaction has however been directly linked to health care outcomes such as use of facilities, expenditure and even mortality [3]. Despite these ambiguities, satisfaction is often used in evaluating and directing the delivery of health care [1]–[7]. The importance attributed to satisfaction, its clinical relevance and the ambiguities in its interpretation and use [8] make the understanding of the determinants of patient satisfaction very relevant to patients, managers and clinicians.

Research into patient satisfaction, amongst older persons, has yielded conflicting findings concerning the roles of age and morbidity as determinants. Overall, older age is found to be related to higher satisfaction [9], [10] and an increase in morbidity and ailments related to lower satisfaction [2], [3], [11]. Since increasing age is related to a higher prevalence of morbidity and ailments, the relation between satisfaction, age and morbidity remains unclear.

This study investigates the relation between satisfaction and patient characteristics in a large population of older persons in primary care. We hypothesized that the sum of somatic, functional, psychological and social problems, expressed as the complexity of health problems, is a powerful determinant of the self-reported level of satisfaction irrespective of age and the individual elements of morbidity. We therefore examined the associations between complexity of health problems, age and individual components of morbidity with satisfaction in older persons in primary care. A strong influence of the complexity of health problems would help to understand the seemingly contradictory finding that increasing age is related to higher satisfaction while the age related increase in morbidity is related to lower satisfaction.

## Methods

### Study population

The current study is embedded in the ISCOPE study (Integrated Systematic Care for Older Persons) in which data on demographic and clinical characteristics of primary care patients aged ≥75 years living in the community and in care homes were obtained.

The overall aim of this study was to assess the efficacy of a simple structural monitoring system to detect deterioration in functional, somatic, mental or social health of individuals aged 75 years and over, followed by the execution of a care plan for those people with a combination of somatic, functional, mental and social problems.

The study population was recruited from 59 participating primary care practices (560 practices were invited). All registered persons aged ≥75 years were targeted (n = 12066). After excluding 590 persons whom were deceased, too ill, non-Dutch speaking, admitted to a nursing home, or not considered suitable by their general practitioner (GP), 11476 persons were sent a written screening questionnaire (Appendix S1). Non-responders were reminded by telephone and if necessary were assisted by research nurses to fill in the screening questionnaires. A total of 7285 screening questionnaires were completed.

Of the older persons returning the screening questionnaire, a random sample was visited at home to obtain data on social and demographic characteristics, and to administer additional questionnaires. Based on the outcomes of the screening questionnaire, all respondents scoring positive in 3 or more domains were approached for an interview. Of those reporting no problems and those scoring problems on 1 domain, a random sample of 15% was interviewed. Of those scoring in 2 domains, a random sample of 60% was interviewed. A total of 2713 interviews was performed at home by trained research assistants and consisted of questions concerning demographics, health and illness and validated questionnaires exploring perceived health, functional limitations, depression, cognition, loneliness, quality of life, healthcare use and satisfaction.

For the present study, 2664 participants with complete data on the question about patient satisfaction were included in the analyses. All participants in the interviews gave written informed consent. The Medical Ethics Committee of the Leiden University Medical Centre approved the study. The study was registered in the Netherlands Trial Register (Registration number 1946).

### Study parameters

#### Satisfaction

The interview included questions about the level of satisfaction the respondent felt with their various care providers including, specifically, the GP practice. In the present study satisfaction was scored on a 5-point Likert scale with the choice options ‘being very satisfied’, ‘satisfied’, ‘neutral’, ‘dissatisfied’ and ‘very dissatisfied’.

Previous research has indicated that, for patients, the choices ‘very satisfied’ and ‘satisfied’ are very different: i.e. ‘very satisfied’ is considered a clear cut above the expected whereas ‘satisfied’ is associated with average care, i.e. more or less adequate. [12] ‘Dissatisfied’ and ‘very dissatisfied’ are regarded as a negative choice. Therefore, we regrouped the five answers to the satisfaction questions into three categories, i.e. Satisfied ( =  very satisfied), Neutral ( =  satisfied and neutral) and Dissatisfied ( =  dissatisfied and very dissatisfied). For the purpose of the logistic regression analysis, satisfaction was also dichotomized into two groups, i.e. Satisfied (including very satisfied, satisfied and neutral), and Dissatisfied (including dissatisfied and very dissatisfied).

#### Complexity of health problems

The term complexity is used widely in medical literature, amongst others in the context of complexity science [13]. In this study, complexity of health problems is seen as a characteristic of an individual patient, describing his or her health- and care situation. We operationalized complexity of health problems as the number of domains (somatic, functional, psychological, social), in the ISCOPE screening questionnaire, with two or more positive answers (Appendix S1).

Each domain included 4–9 questions, derived from existing validated questionnaires [14]–[16].

The respondents were categorized into five groups, ranging from problems in 0 domains to problems in 4 domains.

#### Sociodemographic characteristics

Data on sociodemographic characteristics age, gender, country of birth, level of education and living situation were obtained. Education level was categorized based on the highest completed level of education. Living situation was registered as being either in the community or a residential home.

#### Functional status

Functional status was measured with the Groningen Activities Restriction Scale (GARS) [14], which provides an overall score for limitations in the activities of daily living (ADL). The questionnaire consists of 18 questions. Questions were phrased as: ‘Can you fully independently,…?’, answers range from ‘Without any difficulty’ (1 point) to ‘Not fully independently with someone's help’ (4 points). The overall score ranges from 18–72 with a higher score indicating more severe restrictions.

#### Health and illness

Self-perceived health was scored using a visual analogue scale (VAS) with 0 as the lowest possible level and 100 as the best imaginable level. Self-reported diseases and ailments were obtained during the interview which were grouped within the following 19 chronic diseases: diabetes, heart failure, malignancy, chronic obstructive pulmonary disease, incontinence, arthritis, osteoporosis, dizziness, lower urinary tract symptoms, depression, anxiety, dementia, vision, deafness, fracture, stroke/transient ischemic attack and myocardial infarction.

#### Psychological

The Mini Mental State Examination (MMSE) provides a measure for cognitive impairment and ranges from 0 (very impaired) to 30 (not impaired) [17].

The Geriatric Depression Scale 15 items (GDS-15) provides a measure for the presence of depressive symptoms, specifically for the elderly, ranging from 0 to 15 (not depressed to depressed) [18]. The GDS-15 was obtained only from participants who had an MMSE score ≥18 points.

#### Social

The Loneliness Scale of De Jong Gierveld (DJG) provides a score for loneliness encompassing both emotional and social loneliness, on an 11-item scale, with higher scores indicating more severe loneliness. [16] This loneliness scale was restricted to people with an MMSE score ≥19.

Quality of life was measured with the Dutch EQ5D scale and is expressed as a number, with a maximum of 1.0 indicating optimal quality. Cantril's ladder is a VAS, ranging from 0 to 10, in which the respondent indicates his/her perceived quality of life at this moment (10 being the best imaginable).

### Statistical analysis

Categorical variables were expressed in percentages. Differences between groups in categorical variables were analyzed using Pearson's Chi-square test. Continuous variables were expressed as median and interquartile range. Differences between groups in continuous variables were analyzed with the Kruskal-Wallis test.

The association between complexity and satisfaction was investigated with logistic regression models. We constructed three subsequent regression models. In the first model, crude odds ratios (OR) for the relation between complexity and satisfaction were estimated. The second model was an extension of the first by adjusting for age. The third model included additional adjustments for gender, living situation, disability in daily living (GARS score), number of diseases, cognitive function (MMSE score), subjective health (VAS), quality of life (EQ5D, Cantril score), depressive symptoms (GDS-15 score) and loneliness (DJG). For stability of the logistic regression models, GDS-15 [low (≥4) and high (≥5)] [19] and DJG were dichotomized [low (≤3) and high (≥3)].

Analyses were conducted with IBM SPSS version 20.

## Results

The study population had a median age of 82 (IQR 79-87) years and was predominantly female (68%), of Dutch ethnicity (91%), community dwelling (89%) and had an education level higher than primary school (34% primary school only).

Most participants were satisfied with their GP practice; (very satisfied 37.3%, satisfied 49.9%, neutral 8.7%, dissatisfied 3.4%, very dissatisfied 0.7%). This predominance of satisfaction was also present when the level of satisfaction was divided into the three categories (satisfied 37.3%, neutral 58.6%, dissatisfied 4.1%).

No age differences were found between the three satisfaction categories. The median age for participants in the satisfied group was 82 (IQR 79-87) years, compared with 83 (IQR 79-87) years in the neutral group and 83 (IQR 79-88) years in the dissatisfied group (Kruskal-Wallis; p = 0.140)

Between the three satisfaction categories, no differences were observed in gender (p = 0.271), country of birth (p = 0.353) or education level (p = 0.248). Significant differences were found for living situation; the percentage living in a residential home was significantly higher in the dissatisfied group (p = 0.003) than in the neutral and satisfied group (19% vs 11% vs 9%; p for trend = 0.003). No other associations between demographic characteristics and satisfaction were found. Satisfaction correlated with all of the clinical characteristics; a lower satisfaction level was associated with poorer performance on all test characteristics and with a greater number of diseases.

**Figure 1:**
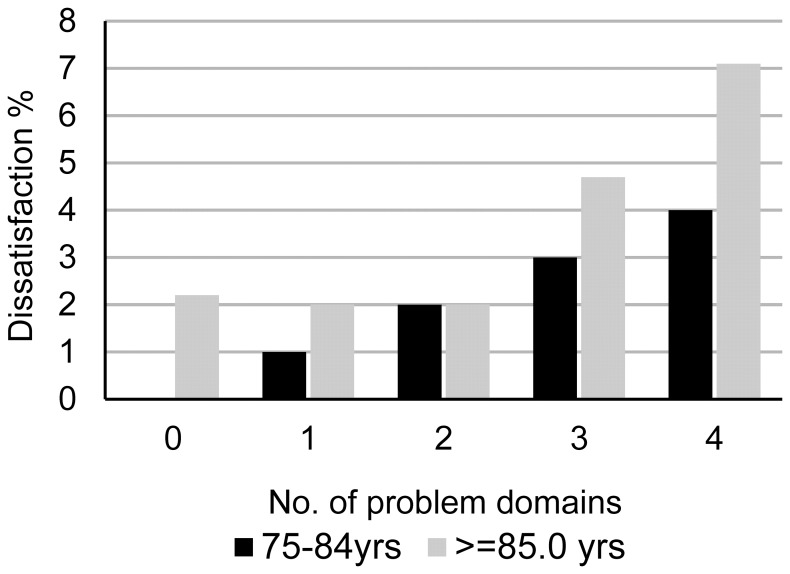
Dissatisfaction in relation to the number of problem domains and age.

The level of satisfaction was inversely associated with the complexity of health problems (Table 1) (p<0.001). Satisfaction was similar between participants with 0 and 4 problem domains (i.e. 11% and 15%, respectively) whereas dissatisfaction showed considerable variation (1% and 34%, respectively). Figure 1 shows the association between the percentage of dissatisfied participants and the number of problem domains for the groups aged ≤ and ≥85 years. In both age groups there was increased dissatisfaction with an increasing number of problem domains.

**Table 1:** Demographic and clinical characteristics related to level of satisfaction of older persons with the general practice.

				Total population	Level of satisfaction			
					Satisfied	Neutral	Dissatisfied	p-value
				n = 2664	n = 994	n = 1561	n = 109	
					(37.3%)	(58.6%)	(4.1%)	
**Sociodemographic characteristics**								
Age		Years		82 (79–87)	82(79–87)	83(79–87)	83(79–88)	0.140
Gender		Female		1819 (68%)	66%	69%	69%	0.271
Place of birth		Netherlands		2427 (91%)	90%	92%	89%	0.353
Education level		Primary only		962 (36%)	34%	37%	40%	0.248
Living situation		Community		2381 (89%)	91%	89%	81%	0.003
**Functional and clinical characteristics**								
Activities of daily living		GARS (points)		32 (24–41)	30 (24–39)	32 (24–42)	38 (31–46)	<0.001
Number of diseases		Sum max. 19		4 (3–6)	4 (3–6)	4 (3–6)	5 (3–6)	0.005
Subjective health		VAS		70 (55–75)	70 (55–75)	70 (55–75)	60 (50–70)	<0.001
Depression		GDS		2 (0–4)	1 (0–3)	2 (0–4)	3 (1–6)	<0.001
Cognitive function		MMSE		28 (26–29)	28 (26–29)	28 (26–29)	27 (25–29)	<0.001
Loneliness		DJG		2 (0–5)	2 (0–4)	3 (1–5)	4 (2–7)	<0.001
Quality of life		EQ5D Dutch tariff		0.8 (0.5–0.8)	0.8 (0.6–0.8)	0.8 (0.6–0.8)	0.5 (0.2–0.7)	<0.001
		Cantril's ladder		7 (7–8)	6 (7–8)	7 (7–8)	7 (6–8)	<0.001
**Complexity of health problems**								
0 problem domains				243 (9%)	11%	9%	1%	<0.001
1 problem domain				212 (8%)	9%	8%	4%	
2 problem domains				726 (27%)	29%	27%	21%	
3 problem domains				1013 (38%)	37%	39%	40%	
4 problem domains				461 (17%)	15%	18%	34%	

Categorical data are represented as n (%). Differences were tested with Chi-square tests. Numerical data are presented as median (IQR). Differences were tested with Kruskal-Wallis tests.

Table 2 shows the crude and adjusted ORs of being dissatisfied with the care provided by the GP practice, related to the complexity of health problems. The risk of being dissatisfied increased with an increasing number of complex health problems. Compared to those without problems, this risk of being dissatisfied increased from 4.6 (95% CI 0.5–42) for participants with 1 problem to 21 (95% CI 2.9–155) for participants with health problems on 4 domains. Per additional problem domain, the risk of dissatisfaction increased 1.7 times (OR 1.7 95% CI 1.4–2.2; Ptrend <0.001). This association remained similar when adjusted for age (1.7 95% CI 1.4–2.2; Ptrend <0.001). When adjusted for age, gender, living situation, disability in daily living (GARS score), number of diseases, MMSE, VAS, EQ5NL, Cantril, GDS-15 and DJG the association remained present (OR 1.4, 95% CI 1.1–1.8; Ptrend = 0.021).

**Table 2:** Risk of older persons to be dissatisfied with GP care related to complexity of health problems, with adjustment for age.

Number of domains	Crude			Adjusted for age		
	OR	95% CI	p-value	OR	CI	p-value
0	1			1		
1	4.6	0.5–42	0.171	4.6	0.5–42	0.171
2	7.9	1.1–59	0.043	7.9	1.1–59	0.040
3	11	1.5–80	0.018	11	1.5–80	0.018
4	21	2.9–154	0.003	21	2.8–154	0.003
Per domain increase	1.7	1.4–2.2	<0.001	1.7	1.4–2.2	<0.001

## Discussion

In the present study on older persons in primary care, the level of patient satisfaction was not associated with age or other demographic characteristics. However, the complexity of health problems of older persons was associated with lower satisfaction, independent of age, gender, living situation, functional status, number of diseases, cognitive impairment, self-perceived health, quality of life, depression and/or loneliness.

When exploring the association between the number of problem domains and the level of satisfaction, the expressed ‘dissatisfaction’ showed more variation compared with ‘satisfaction’.Interestingly, there was a higher frequency of satisfaction in the group with 0 problem domains. This frequency decreased and gradually transformed into a predominance of dissatisfaction in the group with 4 or more problem domains. This suggests that the positive relation between increasing age and satisfaction reported by others [9], [10] may only hold true for groups with a low complexity of health problems. This association is no longer present with a higher complexity load. These findings may indicate that a heavier load of care complexity leads to a lower level of satisfaction with GP care and that this relation is primarily related to the complexity load and not to age, demographics or one of the individual aspects of morbidity. This confirms our initial hypothesis that the complexity of health problems is more strongly associated with the level of satisfaction than age and/or demographic and clinical parameters.

As patient satisfaction is used as an outcome in care evaluation and is a goal of care organisation in itself, understanding its meaning is relevant. Our findings make the following contributions. First, when investigating the relation between individual patient characteristics and satisfaction, the complexity of health problems of the elderly persons must be taken into account. Having shown that the complexity is a stronger determinant than the individual characteristics, the mean complexity level of the population from which the sample is drawn could distort conclusions attributed to individual characteristics. Second, where the complexity load is greatest and therefore the demands on the health system are largest, this negative influence on the level of satisfaction by older users is strongest. This effect should be taken into account when using satisfaction in evaluating care organization and delivery. Third, we found dissatisfaction to be a relatively infrequent but meaningful indication of the level of satisfaction as demonstrated by the high odds ratios in table 2, the confidence intervals for the groups with 3 and 4 complexity domains, although wide, having lower limits well above 1.

Our study shows that a relatively large population is necessary to study satisfaction and draw conclusions with statistical significance. This is due to the inherently high levels of satisfaction allowing limited room for change and expressions of dissatisfaction. We think therefore that, although the statistical power is a challenge for researchers, in the daily situation, practitioners and managers should pay attention to changes in expressions of satisfaction and particularly dissatisfaction in older patients.

A strength of the present study is the large population of older people in primary care, recruited from a range of GP practices, providing a representative group of older persons in primary care regarding age, morbidity and complexity of health problems. In contrast to other studies, high levels of morbidity and the presence of complex health problems were not a reason for exclusion in our study. This enabled us to examine the relation between satisfaction with GP practice, age and complexity of health problems in a representative sample of older persons in primary care. Since the number of persons that indicated being dissatisfied with the provided care was relatively small, we were unable to perform in-depth analyses in smaller subgroups.

In conclusion, among these older persons, satisfaction with the GP practice does not increase with age. However, dissatisfaction with the GP practice is strongly correlated with higher levels of complexity of health problems, independent of age and/or demographic and clinical parameters. It remains unclear whether the complexity of health problems is a patient characteristic influencing the perception of the offered care, or whether the primary care offered is unable to handle the demands of patients with complex healthcare problems, resulting in a lower level of satisfaction.

Further unravelling of the relation between satisfaction, complexity of health problems and the individual constituents of morbidity, such as depression and loneliness, is necessary. Also prospective studies are needed to investigate the causal associations between care organization and delivery, patient characteristics, indicators of quality, and patient perceptions.

## Supporting Information

Appendix S1 **ISCOPE screening questionnaire.**
(DOC)
